# Surface Functionalization of Ureteral Stents-Based Polyurethane: Engineering Antibacterial Coatings

**DOI:** 10.3390/ma15051676

**Published:** 2022-02-23

**Authors:** Kardelen Ecevit, Eduardo Silva, Luísa C. Rodrigues, Ivo Aroso, Alexandre A. Barros, Joana M. Silva, Rui L. Reis

**Affiliations:** 1 3B’s Research Group, I3Bs–Research Institute on Biomaterials, Biodegradables and Biomimetics, University of Minho, Headquarters of the European Institute of Excellence on Tissue Engineering and Regenerative Medicine, Avepark, Parque de Ciência e Tecnologia, Zona Industrial da Gandra, 4805-017 Barco GMR, Portugal; kardelen.ecevit@i3bs.uminho.pt (K.E.); eduardo.silva@I3Bs.uminho.pt (E.S.); luisa.rodrigues@i3bs.uminho.pt (L.C.R.); ivo.aroso@i3bs.uminho.pt (I.A.); ip@i3bs.uminho.pt (A.A.B.); rgreis@i3bs.uminho.pt (R.L.R.); 2 ICVS/3B’s PT Government Associated Laboratory, 4805-017 Guimaraes, Portugal

**Keywords:** surface modification, natural compounds, chitosan, fatty acids, antimicrobial, biofilm, ureteral stent

## Abstract

Bacterial colonization of polyurethane (PU) ureteral stents usually leads to severe and challenging clinical complications. As such, there is an increasing demand for an effective response to this unmet medical challenge. In this study, we offer a strategy based on the functionalization of PU stents with chitosan-fatty acid (CS-FA) derivatives to prevent bacterial colonization. Three different fatty acids (FAs), namely stearic acid (SA), oleic acid (OA), and linoleic acid (LinA), were successfully grafted onto chitosan (CS) polymeric chains. Afterwards, CS-FA derivatives-based solutions were coated on the surface of PU stents. The biological performance of the modified PU stents was evaluated against the L929 cell line, confirming negligible cytotoxicity of the developed coating formulations. The antibacterial potential of coated PU stents was also evaluated against several microorganisms. The obtained data indicate that the base material already presents an adequate performance against *Staphylococcus aureus*, which slightly improved with the coating. However, the performance of the PU stents against Gram-negative bacteria was markedly increased with the surface functionalization approach herein used. As a result, this study reveals the potential use of CS-FA derivatives for surface functionalization of ureteral PU stents and allows for conjecture on its successful application in other biomedical devices.

## 1. Introduction

Ureteral stents are commonly used to treat neurogenic bladder, urinary flow obstruction (kidney stones and obstructive tumors), or acute urinary retention to provide urine drainage from the kidney to the bladder [1]. However, although these indwelling ureteral stents offer practical solutions in various clinical situations, their applications may result in severe complications such as infection and bacterial colonization on the surface of stents due to non-sterile urine, which contains complicated microbial communities even in healthy or asymptomatic cases [2]. Inevitably, planktonic microorganisms in the urine are prone to adhere and colonize on the surface of these indwelling devices. This behavior of microorganisms provokes the formation of resistant biofilms and, consequently, infections [3]. As reported by the National Healthcare Safety Network (NHSN), the use of ureteral stents accounts for nearly 75% of hospital-acquired urinary tract infections as a consequence of pathogen adhesion to the medical device surface [4,5]. Some of these infections are caused by biofilm formation comprising microbial communities resistant to commonly applied antibiotics, posing a serious health hazard to patients [3]. Recently, several studies reported that microorganisms within biofilms show greater antibiotic resistance compared to the ones in the planktonic state, requiring concentrations 10–1000 times greater to display a similar effect [6]. Therefore, increasing concern about the critical complications associated with the use of these devices and the wide spread of antibiotic resistance has led to a concerted effort by the scientific community to develop functional solutions to the problems associated with these medical devices.

The development of therapeutic strategies for the prevention of infections has focused on mitigating bacterial adhesion on the surface of indwelling devices. In line with this focus, several coating methodologies have been extensively studied, such as immobilizing antimicrobial agents onto substrate material by physical adsorption or covalent bonding, and the inclusion of the antimicrobial agent within the substrate material [7,8,9]. Another critical issue is the selection of the ideal coating agent, which should virtually present antimicrobial efficacy, be properly functionalized onto the stent surface, possess desirable biocompatibility, and be cost-effective [1].

To date, numerous inorganic and organic-derived antibacterial agents have been discovered and utilized in various applications, including catalysis, medical devices, water treatment, drug delivery systems, cosmetics, catalysts, food processing, biosensors, filtration technologies, gene therapy, and tissue engineering applications [10,11]. Among these applications, the development of medical devices with antibacterial properties has become an urgent need in combating bacterial infection and its emerging vital outcomes. Therefore, recent approaches have focused on combining surface modification and coating methods to develop novel materials using a range of different raw materials such as hydrophilic and hydrophobic polymers, antibiotics, nanoparticles, biodegradable materials, antifouling agents, quorum-sensing signaling molecules and bacteriophages, etc. [12].

Although these strategies improve in vitro antimicrobial performance of these devices, they are also confronted by considerable barriers, limiting their clinical use. These limitations include high cytotoxicity, short-term antimicrobial activity, effectiveness against a limited spectrum of bacterial strains, and acquired and/or developed bacterial resistance. Confronted by these obstacles, natural compounds hold promise with their featured properties such as broad-scale structural diversity, antimicrobial mechanisms through non-specific interaction against a large spectrum of pathogens, interference with multiple targets, rarer side-effects, and relatively low costs [13]. Hence, the bioactivity of natural compound-based coating materials on polymeric surfaces has been widely recognized in recent years, providing vital opportunities in the development of antimicrobial performance.

Fatty acids (FAs), commonly obtained from vegetable oils or animal fat, are attracting widespread interest as promising antimicrobial agents [14]. Their chemical structure consists of carboxylic acids with long and unbranched aliphatic chains that can possess different saturation degrees. Along with their various biological activities, in the past few years, the antibacterial efficiency of FAs towards many pathogenic microorganism species has generated considerable research interest [15,16]. The mechanism of action of these bioactive compounds, which makes them stand out in the fight against antibacterial resistance, consists of their acting upon multiple sites leading to several effects, such as disruption of the cell membrane, interference with cellular energy supply, the prevention of enzyme activities, impaired nutrient uptake, direct bacterial cell lysis or formation of degradation products by peroxidation and auto-oxidation [17].

Similarly, chitosan (CS) and its derivatives are well-known and commonly used compounds due to their prominent biological features, such as biodegradability, biocompatibility, and low immunogenicity [18]. Nevertheless, numerous modifications or grafting of the CS side chain have been attempted, including with FAs, in order to increase biological efficiency. For example, an in vitro study on the potential use of nonviral gene delivery vectors of eight different saturated FAs grafted CS polymers and their nanomicelles confirmed that CS led to relatively lower cell viability compared to CS-FA polymers, and their micelles had negligible cytotoxicity [19]. In another study with CS-FA-based coatings, CS was grafted onto three different FA derivatives (linoleic acid, α-linolenic acid, and dilinoleic acid) and these CS-FA derivatives were coated onto a poly (ethylene terephthalate)-based substrate. A bacterial colonization assay showed that CS, CS-linoleic acid, and CS-α-linolenic acid-based coatings reduced *E. coli* colonization by 80%. However, CS-dilinoleic acid-coatings did not prevent *E. coli* colonization [20]. Additionally, previous studies have emphasized that various FAs and their derivatives exhibit antimicrobial activity against several bacterial targets through a broad range of direct and indirect inhibitory effects, depending on their chain length and saturation degree [21,22,23].

In urinary environments, the material composition of the medical devices, as well as stent design and applied coatings, are critical considerations to eradicate or reduce stent-related complications. In ureteral stents, the main raw material is generally constituted by polymeric compounds such as silicone, polyurethane (PU), polyethylene, polytetrafluoroethylene, and polyvinyl chloride [24]. PU-based urinary stents became prominent due to their inert structure, good shape memory, low cost, remarkable mechanical properties and biocompatibility [25]. However, the main drawback of PU stents is their hydrophobic surface, which is conducive to protein adsorption and, thus, promotes bacterial adhesion and deposition of inorganic compounds [26]. CS-immobilized PU materials have been reported to show potent antibacterial activity; however, the antibacterial effect of CS may not be sufficient in urinary stents contacting urine harboring complex microbial communities. Thus, conjugation of CS with FA derivatives, which act via different mechanisms of action against a broad range of bacteria, as previously explained, possesses promising potential as a coating material in ureteral stents. Even though studies on the synthesis and characterization of such CS-FA derivatives have already been reported, to the best of our knowledge, this is the first time their antibacterial potency as a coating material on ureteral stents-based PU has been evaluated.

Considering these facts, the main purposes of this study included the effective fabrication of CS-FA derivatives-coated PU stents, following the synthesis of CS-FA derivatives by a straightforward strategy, and ultimately evaluating their biological performance. Therefore, this study intensely focused on scrutinizing the anti-bacterial performance of PU stents coated with CS-FA derivatives against several Gram-positive and Gram-negative bacteria. Accordingly, CS-FA derivatives based on SA, OA, and LinA with the same chain length and different degrees of saturation were selected. Hereby, this study reveals clues and pertinent knowledge on the potential of CS-FA derivatives for surface functionalization of ureteral PU stents and may open the road to its successful application in other biomedical devices.

## 2. Materials and Methods

### 2.1. Grafting of FA Derivatives on CS

CS (Molecular weight 40–150 kDA, 95/20, Heppe Medical Chitosan GmbH, CAS Number 9012-76-4, Halle (Saale), Germany) was grafted with three different FA derivatives, including either SA (Sigma-Aldrich, CAS Number 57-11-4, New Road, Gillingham, Dorset, UK), OA (90–100%, MP Biomedicals, CAS Number 112-80-1, Solon, OH, USA) or LinA (58–74%, Sigma-Aldrich, CAS Number 60-33-3, Algés, Portugal) in the presence of EDC (EDC·HCl, Sigma-Aldrich, CAS Number 25952-53-8, Saint Louis, MO, USA) and NHS (Sigma-Aldrich, CAS Number 6066-82-6, Saint Louis, MO, USA). CS-FA derivatives were protected from light to prevent the oxidation of FAs. Summarily, CS (1 g) was dissolved in 66.6 mL of a 1% *v*/*v* acetic acid solution (99, 8%, puriss. p.a Sigma-Aldrich, CAS Number 64-19-7, New Road, Gillingham, Dorset, UK) and then diluted to 100 mL with methanol (puriss. p.a., Sigma-Aldrich, CAS Number 67-56-1, New Road, Gillingham, Dorset, UK). The pH of the solution was adjusted to 5.2 with 0.1 M sodium hydroxide solution (Panreac Applichem, CAT Number 131687, Barcelona, Spain). The synthesis process was carried out using a weight ratio of each FA relative to CS by fixing at 0.17. FA derivatives were dissolved in 15 mL of ethanol for SA and methanol for OA, and LinA for 2 h at RT followed by the addition of EDC (3 mol/mol of FA). After 20 min, NHS (3 mol/mol of FA) was added to the mixture of FA and EDC, and the resultant mixture was dropwise added to CS solution under constant stirring at RT. The reaction was conducted at RT for 24 h at a pH range of 5–6. The ultimate product was obtained by precipitating with 200 mL of methanol/ammonia solution (Honeywell Fluka, Product number 05002-10185738, Seelze, Germany) (7:3 *v*/*v*), centrifuged, washed with methanol and deionized water, and freeze-dried. Obtained products were stored at 4 °C and protected from light.

### 2.2. Fabrication of CS-FA-Coated PU Stents

PU stents (Nordson Tecoflex, RR0603WH1B) were supplied by HydrUStent (Guimarães, Portugal) and chosen as substrate material since they are employed to manufacture various urological devices. PU stents were cut into pieces (3 cm), ultrasonically cleaned in isopropyl alcohol (Honeywell Riedel-de Haën™, Product number 33539, Seelze, Germany) and methanol twice for 15 min, and dried at RT. The introduction of carboxyl groups on the surface of PU was performed using a previously reported approach [27,28]. Briefly, Jone’s reagent was prepared by dissolving 7 g of chromium (VI) oxide (CrO_3_), for synthesis, Merck, CAS Number 1333-82-0, Darmstadt, Germany) in 500 mL of deionized water and then slowly adding 65 g of concentrated sulfuric acid (H_2_SO_4_, 95–97%, puriss pa, Sigma-Aldrich, CAS Number 7664-93-9, Seelze, Germany). After adding H_2_SO_4_, the solution was removed from an ice-water bath and mixed continuously until RT was achieved. The above cleaned PU stents were treated with 5 mL of Jone’s reagent at 160 rpm for 30 min at RT in the dark and washed with deionized water twice. PU stents treated by Jone’s reagent (PU-J) were immersed in 300 mL of a 30 wt.% aqueous acrylic acid solution (stabilized with hydroquinone monomethyl ether, Sigma-Aldrich, CAS Number 79-10-7, Darmstadt, Germany) containing 0.1 mL 0.015 M Iron(II) sulfate heptahydrate (FeSO_4_, Sigma-Aldrich, ≥99.0%, CAS number 7782-63-0, Saint Louis, MO, USA)/0.005 M H_2_SO_4_ in a round bottom flask under nitrogen gas to fulfill graft polymerization at 45 °C for 150 min. Afterwards, the samples were rinsed with deionized water, and dried at 40 °C under vacuum [29]. PU stents enriched in carboxyl groups were immersed in MES buffer (the pH 5.0–6.0, Sigma-Aldrich, CAS Number 1266615-59-1) for 30 min. Then, each piece of PU-AAc was incubated in 2 mL of EDC/NHS (0.1 M EDC, 0.2 M NHS) in MES buffer (the pH 5.0–6.0, Sigma-Aldrich, CAS Number 1266615-59-1, Saint Louis, MO, USA) at 4 °C for 2 h in the dark. After washing activated PU stents with MES buffer, PU stents were immersed in CS-FA derivative solution (5 and 10 mg/mL). Subsequently, the samples were rinsed with deionized water and air-dried.

### 2.3. Physicochemical Characterization

#### 2.3.1. Fourier Transform Infra-Red in Attenuated Total Reflection Mode–(FTIR-ATR)

Commercial CS, CS-FA derivatives, CS and CS-FA derivatives-coated PU stents were analyzed by FTIR-ATR spectroscopy. FTIR-ATR measurements were performed employing an IR-Prestige-21 spectrophotometer (Shimadzu Scientific Instruments, Columbia, MD, USA) by averaging 32 individual scans over the spectral range from 4000–500 cm^−1^ with a resolution of 4 cm^−1^.

#### 2.3.2. Proton Nuclear Magnetic Resonance (^1^H-NMR)

NMR experiments were performed with a 400 MHz Bruker Advance II (Bruker, Rheinstetten, Germany). In addition, Mestrenova 12.0 software (Mestrelab Research, Santiago, Spain) was employed for spectral treatment. Methanol-d4 (≥99.8 atom % D, Sigma-Aldrich, CAS Number 811-98-3, Saint Louis, MO, USA), acetic acid-d4 (≥99.5 atom % D, Sigma-Aldrich, CAS Number 1186-52-3, Saint Louis, MO, USA), deuterium oxide (99.9 atom % D, Sigma-Aldrich, CAS Number 7789-20-0, Saint Louis, MO, USA) and dimethyl sulfoxide-d6 (DMSO-d6, 99. atom % D, Euriso-top, CAS Number 2206-27-1, St. Aubin, France) were used as received.

Synthesized CS-FA derivatives were dissolved (5 mg/mL) in a mixture of 2% acetic acid-d4 in deuterium oxide and methanol-d4 (3:7 *v*/*v*). For analyzing the base materials, a commercial CS sample (10 mg/mL) was prepared in deuterium oxide containing 2% acetic acid-d4. Commercial FA derivatives were prepared at a concentration of 10 mg/mL in dimethyl sulfoxide-d6. The spectrum of commercial FA and CS-FA derivatives were recorded at RT, while for commercial CS, 70 °C was used. All the experiments were conducted while the systems were in equilibrium, and no additional abnormality in their features was detected.

DS of N-substituted CS-FA derivatives was evaluated by comparing the integration ratios of terminal methyl protons (H18) of FA derivatives (δ = 0.90 ppm) to H2 proton of GlcN monomer in CS (δ = 3.04 ppm), as represented by Equation (1) [30,31,32]:

(1) DS(%)=ICH33IH2×100

where three is the number of protons from the signal of the methyl proton, and I_H-2_ is the integral for the single proton of the H2 position on the CS.

#### 2.3.3. X-rays Photoelectron Spectroscopy (XPS)

The samples were analyzed utilizing a Kratos Axis-Supra instrument (Kratos Analytical, Manchester, UK) equipped with aluminum Kα (Al-Kα) monochromatized radiation X-ray source at 1486.6 eV, with the assistance of ESCApe software (Version 1.4, Kratos Analytical, Manchester, UK). Photoelectrons were collected from a take-off angle of 90° relative to the sample surface. The chemical composition of samples surfaces was examined in a Constant Analyser Energy mode (CAE) (Kratos Analytical, Manchester, UK) with a 160 eV pass energy for survey spectra and 20 eV pass energy for high resolution spectra and a emission current of 15mA for all the acquisitions. The high-resolution spectra were performed for C 1s, O 1s and N 1s in order to determine the relative composition of the sample’s surface. Charge referencing was done by setting the lower binding energy C 1s photo peak at 285.0 eV, which is related to the C 1s hydrocarbon peak.

#### 2.3.4. Differential Scanning Calorimetry (DSC)

The DSC experiments were performed in a TA instrument DSC Q100 model (Thermal analysis & analyzers, New Castle, DE, USA). Approximately 2.5 mg of each sample was placed into an aluminum pan and installed in the equipment under a purging nitrogen atmosphere (50 mL min^−1^), with an empty aluminum pan as an inert reference. The temperature ramp contained a heating step from 0 °C to 350 °C (heating rate 10 °C min^−1^), and subsequently an isothermal step of 2 min before cooling down the system to 0 °C. The DSC scans were evaluated using Origin Pro 2016 software (Version 9.3.226, OriginLab Corporation, Northampton, MA, USA).

#### 2.3.5. Crystal Violet Staining

Coated PU stents and untreated PU stents were cut into 5 mm lengths. Each sample was cut lengthways with a blade, stained with 1 mL of 0.1% crystal violet (≥90, Sigma-Aldrich, CAS Number 548-62-9, Saint Louis, MO, USA) in deionized water at RT for 10 min, and rinsed with deionized water. CS acts as an efficient adsorbent for crystal violet because of the amino functional groups. After the incubation of samples with crystal violet dye, the surface images of control PU stent, CS and CS-FA derivatives-coated PU stents were photographed by a Stereo Microscope Stemi 1000 (Zeiss, Jena, Germany). The purple color of the coated PU stents confirmed the presence of coatings.

#### 2.3.6. Scanning Electron Microscopy (SEM)

The morphology of the coated PU stents and bacteria colonization on the surface of untreated and the coated PU stents were visualized using the Scanning Electron Microscope (SEM, JSM-6010 LV, JEOL, Tokyo, Japan). Before analysis, all the samples were covered with a conductive gold layer by a sputter coater EM ACE600 (Leica Microsystems, Vienna, Austria).

### 2.4. Cytotoxicity Studies against L929 Murine Fibroblasts

The cytotoxicity of leachables from the untreated (PU) and coated stents against animal cells was assessed using the L929 murine fibroblast ATCC CCL-1™ cell line (American Type Culture Collection, Manassas, VA, USA) according to ISO/EN10993 guidelines. Firstly, 0.5 cm pieces of the different samples were placed in a 1.5 mL microstent containing 1 mL of Dulbecco’s Modified Eagle Medium (DMEM, ref. D5523, Sigma-Aldrich, Saint Louis, MO, USA) and left incubated for 24 h at 37 °C 80 RPM. Posteriorly, 200 µL of the obtained extracts was added to a previously established cell monolayer (10,000 cells/well) in 96-well tissue culture plate and left incubated for 24 h. The following day, possible morphology changes were observed by optical microscopy (Axio Vert A1, Zeiss, Thornwood, NY, USA) and cell viability determined by MTS (ref. G3581, Promega, Madison, WI 53711-5399, USA) colorimetric assay at a 490 nm wavelength. Cell viability (%) for each sample extract was obtained by normalization against cells grown in only DMEM media.

### 2.5. In Vitro Antibacterial Activity

#### 2.5.1. Bacterial Attachment Assay

An AB assay was employed to evaluate the efficiency of preventing the bacterial attachment onto the surface of all CS-FA derivatives-coated-PU stents by using Gram-positive and Gram-negative bacteria, including *S. aureus* ATCC 25923 (American Type Culture Collection, Manassas, VA, USA), MRSA ATCC 700698 (Microbiologics, Inc., Saint Cloud, MN, USA), *E. coli* ATCC 25922 (Microbiologics, Inc., Saint Cloud, MN, USA), and *P. mirabilis* ATCC 51286 (American Type Culture Collection, Manassas, VA, USA) strains, as reported by Horton et al. 2005 [33]. Samples (control PU stent, CS-PU, CS-SA-PU, CS-OA-PU, and CS-LinA-PU) were carefully cut 0.5 cm in length and sterilized with ethylene oxide.

Bacteria strains were inoculated into tryptic soy broth (TSB, Liofilchem, Ref 610053, Roseto degli Abruzzi, Italy) and cultivated overnight by shaking at 160 rpm at 37 °C. The obtained culture was adjusted to an optical density of 0.08–0.1 at 600 nm by diluting with TSB supplemented with 1% D-(+)-glucose (Sigma-Aldrich, CAS Number 50-99-7, Saint Louis, MO, USA). Subsequently, samples were placed into wells of a 48-well polystyrene flat-bottom plate containing 1 mL of the prepared bacterial suspension. Then, the plates were incubated at 37 °C for 24 h at 160 rpm for biofilm formation. Afterwards, non-adherent bacteria were removed by washing twice with 1 mL of Dulbecco’s phosphate-buffered saline (DPBS, Gibco, Catalog number 21600, Grand Island, NY, USA) solution. Subsequently, washed samples were transferred into a 96-well polystyrene flat-bottom, and 200 μL of TSB media including 10%(*v*/*v*) AB dye (Bio-Rad, ref BUF012, Kidlington Oxford, UK) was added. Besides, it should be noted TSB media, including 10%(*v*/*v*) AB, was used as a blank control. Consequently, the plates were incubated at 37 °C 160 rpm for a different period according to bacterial strain, namely, 75 min for *E. coli*, 120 min for *P. mirabilis*, 90 min for *S. aureus,* and 45 min for MRSA. Finally, 100 μL of the solution was transferred to a new microtiter plate for the absorbance detection at 570 and 600 nm using a Synergy HT Microplate Reader (BioTek Instruments, Winooski, VT, USA) to quantify attached bacteria on the surface of samples.

Next, samples were carefully fixed employing a 10% Neutral Buffered Formalin (Thermo Scientific, Ref 5701, Kalamazoo, MI, USA) for 1 h and washed with DPBS solution followed by dehydration via immersion in increasing concentrations of ethanol (20%, 40%, 60%, 80%, and 100%) for 15 min each. Subsequently, samples were dried in a safety cabinet overnight and then gold-coated for SEM image acquisition.

#### 2.5.2. Live/Dead Fluorescence Assay

The LIVE/DEAD^®^ BacLight^TM^ Bacterial Viability Kit (L7007, Invitrogen, Carlsbad, CA, USA) was used to determine the viability of bacterial cells adhered to the surface of obtained samples.

Biofilm formation on the surface of control PU and coated PU stents were provided by employing the same procedure applied in the AB assay. After the incubation step, all the samples were washed individually in 1 mL of DPBS and then stained with 200 μL of BacLight-staining solution (component A and B 1:1) for 20 min at RT in the dark. The BacLight-staining solution was prepared using the LIVE/DEAD^®^ Baclight^TM^ L7007 Kit according to the manufacturer’s protocol. Subsequently, samples were fixed in 10% Neutral Buffered Formalin for 50 min at 4 °C and washed with 1 mL of DPBS followed by image acquisition using a Confocal Laser Scanning Microscope (TCS SP8, Leica Microsystems, Wetzlar, Germany).

### 2.6. Statistical Analysis

Statistical analysis was performed using GraphPad Prism (GraphPad Software Inc., Version 9.1.2, La Jolla, CA, USA) for Windows. The results were statistically analyzed utilizing one-way ANOVA analysis and followed by the use of Tukey’s multiple comparison test at a 5% significance level. A *p*-value < 0.05 was considered statistically significant in all specimens.

## 3. Results and Discussion

### 3.1. Structural Characterization of CS-FA Derivatives

**Figure 1:**
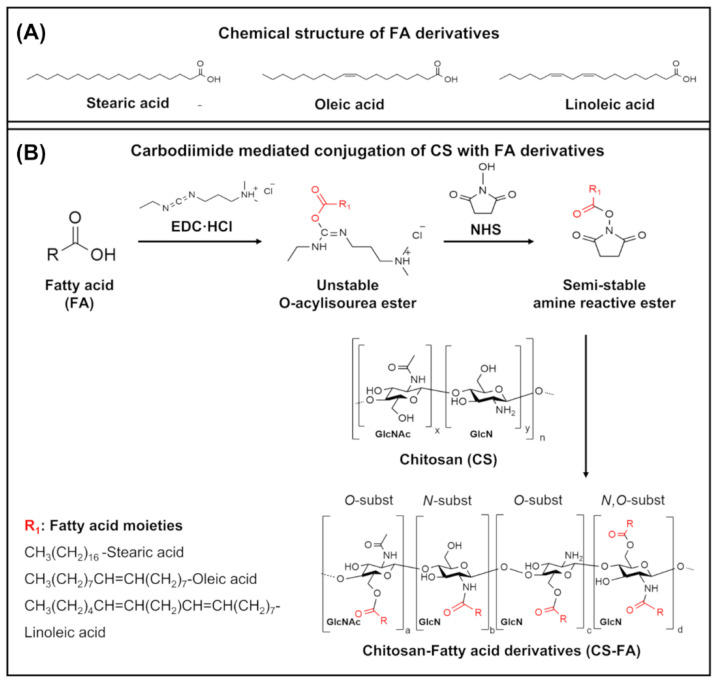
Chemical structure of FA (**A**) Possible reaction products of chemical conjugation mechanism of CS with FA derivatives via carbodiimide mediated coupling reaction (**B**).

**Figure 2:**
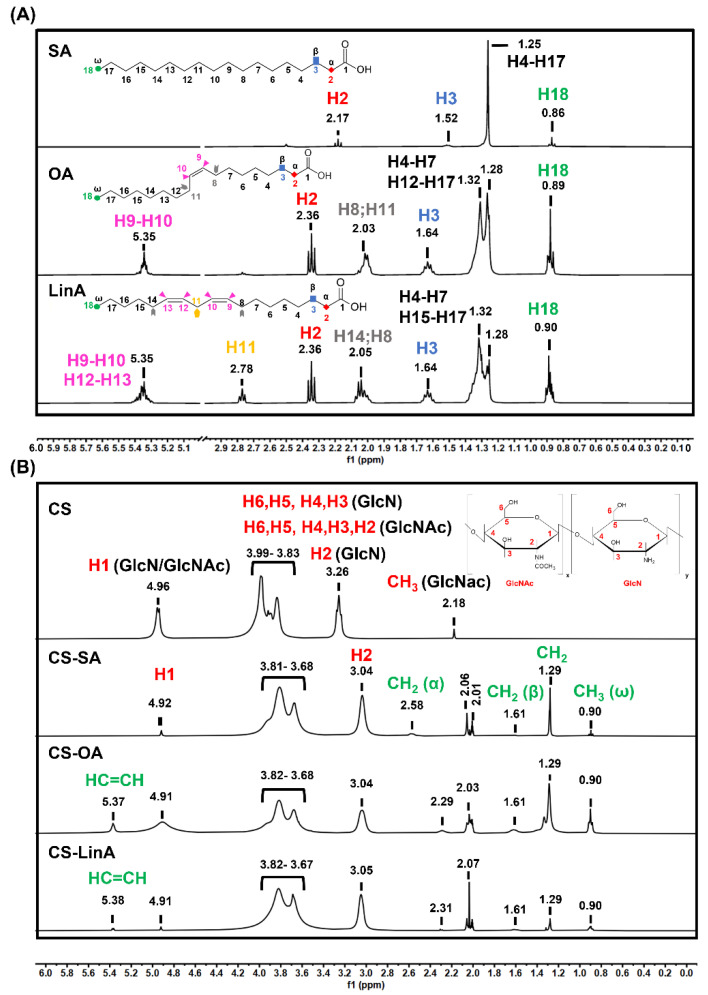
^1^H-NMR spectra of individual FAs (**A**), and CS and CS-FA derivatives (**B**).

The conjugation of CS with different FA derivatives was performed as a modified version of previous studies using different molecular weights and degrees of deacetylation of CS combined with different FAs [19,34]. In this study, a saturated FA (stearic acid, SA) and two distinct unsaturated FAs with varying degrees of saturation (oleic acid, OA, and linoleic acid, LinA) were grafted onto CS; their chemical structures are presented in Figure 1A. Each FA derivative was grafted onto CS via a carbodiimide-mediated coupling reaction using N-(3-Dimethylaminopropyl)-N′-ethylcarbodiimide hydrochloride (EDC) to activate the carboxyl groups by forming a O-acylisourea ester and N-Hydroxysuccinimide (NHS) to convert this ester intermediate into a more stable form as illustrated by Figure 1B. The conjugation reactions of CS-FA derivatives were performed in an acidic environment (pH 5.2), which allows for acylation modification of CS, constituted by a N-acetylglucosamine (GlcNac) and glucosamine (GlcN) unit. Therefore, activated CS in the presence of EDC/NHS can be substituted in an O-acylation at the C6–OH and/or an N-acylation at C2–NH_2_ (see Figure 2B-Chemical structure of CS). Hence, simultaneously N-acylated, O-acylated, and/or N, O-acylated CS derivatives were produced as previously reported [34].

The successful conjugation of CS with FA derivatives was confirmed using Fourier transform infra-red in attenuated total reflection mode-(FTIR-ATR), proton nuclear magnetic resonance (^1^H-NMR), and differential scanning calorimetry (DSC) techniques. NMR spectra were mainly used for structural elucidations and to determine the degree of substitution (DS). The ^1^H-NMR spectra of commercial FAs are presented in Figure 2A, while the ones of commercial CS and CS-FA derivatives are shown in Figure 2B.

By examining the results, it is possible to confirm the purity of SA, OA, and LinA, as the spectra illustrated in Figure 2A confirms prior reports in the literature [34]. The commercial SA spectrum shows the singlet peak ascribed to the long methylene group at δ 1.3 ppm, whereas, in the spectra of commercial OA and LinA (Figure 2A), a well-defined doublet peak was obtained between δ = 1.2–1.4 ppm ascribed to their long methylene group-interrupted double bonds. Additionally, characteristic peaks of FA derivatives were observed at δ 1.06, 1.8, and 2.6 ppm, which are ascribed to protons of terminal methyl (H18), β-methylene (H3), and α-methylene groups (H2), respectively. Besides, the spectrum of commercial CS presents a characteristic peak at δ 2.18 ppm assigned to N-acetyl protons in the GlcNAc monomer (Figure 2B) [34,35]. The protons of H2–H6 of GlcNAc and H3–H6 of GlcN monomers of CS are presented as multiple peaks at δ 3.99–3.83 ppm. Additionally, whereas the peak at δ 4.96 ppm is ascribed to anomeric hydrogen of both GlcNAc and GlcN, H2 proton of the GlcN monomer is located at δ 3.26 ppm [36]. Considering the spectra of CS-FA derivatives, characteristic FA signals of H18, H3, H2 were observed at δ 0.90, 1.61, and 2.59 ppm, respectively. Furthermore, olefinic protons (HC=CH) in CS-OA and CS-LinA can also be observed as multiple peaks at 5.37 ppm [34]. Additionally, it is possible to see that the characteristic proton of CS undergoes chemical shifts at a low field due to conjugation with FA derivatives. This concurs with a structure where the H2 and H6 protons of CS are in interaction with the carbonyl group of FA moieties. Overall, the successful synthesis of the CS-FA is confirmed by the chemical shifts of the CS characteristic signals as presented in the spectra of the CS-FA derivatives. Additionally, NMR data was used to determine the DS (%), which was 2.62% ± 0.72 for CS-SA; 2.37% ± 1.33 for CS-OA; and 3.38% ± 1.74 for CS-LinA. However, it should be noted that only the DS of N-substituted CS-FA derivatives were calculated as the DS of the O-substituted and the N, O-substituted CS-FA derivatives could not be estimated due to the overlapping of the H2 peak of GlcNAc monomers with the pyranose ring protons (H3, H4, H5) and H6 of GlcN monomers in the CS structure.

**Figure 3:**
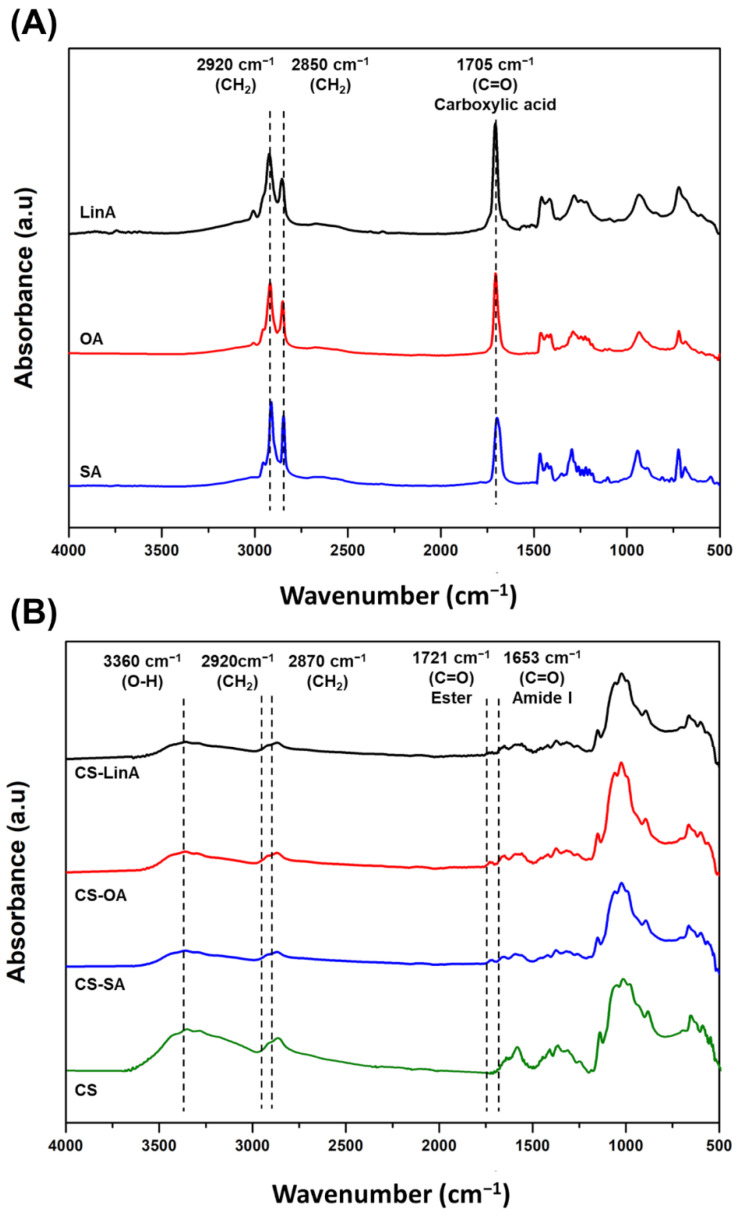
FTIR-ATR spectra of (**A**) commercial FAs, and (**B**) CS-FA derivatives.

FTIR-ATR was also used to confirm the successful grafting of FA derivatives on CS. Figure 3A shows the FTIR-ATR spectra of commercial FAs while commercial CS and CS-FA derivatives are displayed in Figure 3B. CS exhibited a broad peak at the wavenumber range of 3000–3360 cm^−1^, which refers to the O–H stretching vibration [37]. Likewise, CS-FA derivatives (Figure 3B) displayed O–H stretching at the wavenumber range of 3000–3360 cm^−1^. Absorption peaks at 2918 and 2850 cm^−1^ related to –CH_2_ symmetrical and asymmetrical stretching vibrations, sequentially, were superimposed upon –CH_2_ stretching vibrations of commercial CS at 2870 cm^−1^. The FA spectrum (Figure 3A) exhibited a typical band of carboxylic acid at 1705 cm^−1^, which is ascribed to the C=O stretching vibration. In the spectra of the CS-FA derivatives, a new absorption peak at 1721 cm^−1^ is associated with the C=O stretching vibration of the carbonyl ester group of O-substituted CS-FA derivatives. In addition, another peak at 1653 cm^−1^ is ascribed to the C=O stretching vibrations of the amide group in N-substituted CS-FA derivatives [34,36]. The significant advantage of the N,O-acylation reaction pathway is the possibility of obtaining derivatives with the amino group and considerably less reactive hydroxyl groups in the CS structure [38]. Overall, the data suggest that FAs were successfully grafted onto CS through N- or O-acylation reaction.

DSC analysis was also performed to provide insights on the thermal behavior of these CS-FA derivatives, as shown in Figure S1. DSC curves of all samples show an endothermic peak around 63.1 °C ascribed to water evaporation of CS in the heating stage from 0 to 350 °C [39,40,41]. However, in the cooling stage, while the pure CS shows a sharper exothermic peak at 317 °C, indicating the thermal decomposition, the exothermic peaks of the synthesized CS-FA derivatives shifted to lower temperatures than that of the pure CS [42,43]. This result can be correlated with the resulting amide group from the reaction between CS and FA derivatives, consistent with the previous data.

### 3.2. Functionalized PU Stents with CS-FA Derivatives: Physicochemical Characterization

**Figure 4:**
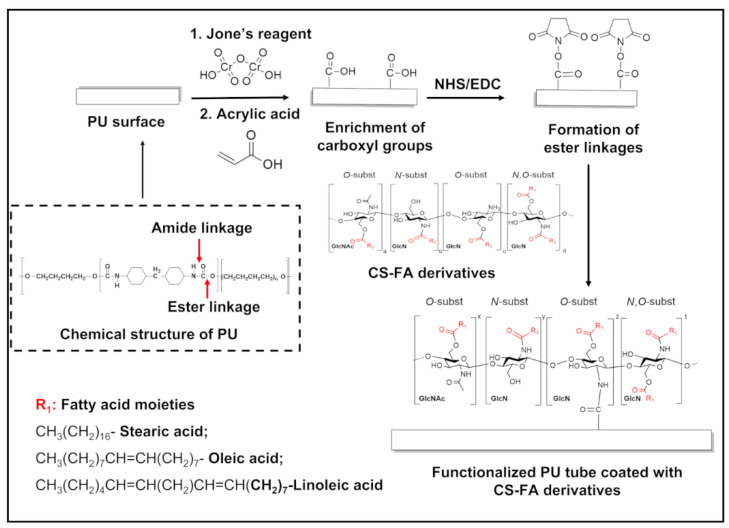
Schematic representation of the modification mechanism of CS and CS-FA derivatives coated onto the surface of PU stents.

**Figure 5:**
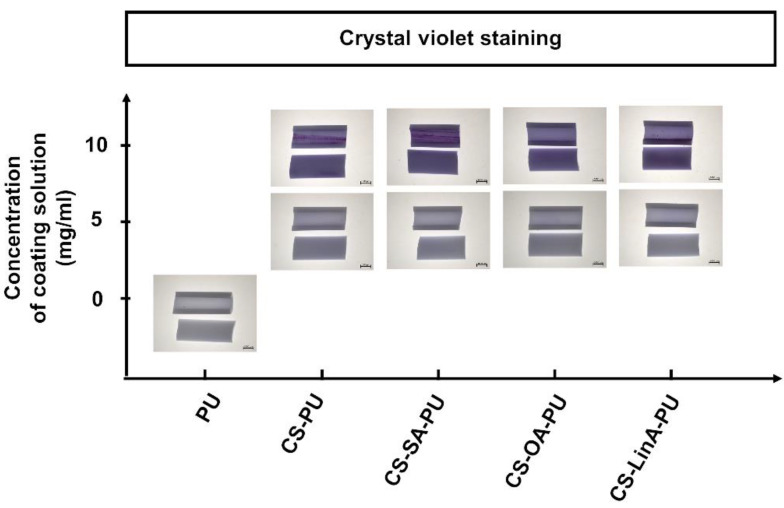
Optical microscopy photograph of untreated PU stent and the coated PU stents at two different concentrations (5 mg/mL and 10 mg/mL) of CS and CS-FA derivatives obtained from the crystal violet staining procedure. (CS-coated PU stent, CS-PU; CS-SA-coated-PU stent, CS-SA-PU; CS-OA-coated-PU stent, CS-OA-PU; CS-LinA-coated-PU stent, CS-LinA-PU).

Surface activation and coating processes were employed to construct the coatings on the surface of PU stents with CS and CS-FA derivatives through covalent interactions. A schematic illustration of each step of the coating procedure undertaken in this study is presented in Figure 4. In general, PU surfaces are treated with Jone’s reagent (CrO_3_/H_2_SO_4_) to generate the carboxyl groups [27,28]. Subsequently, an activation step with acrylic acid (AAc) treatment is used to enrich further the number of carboxyl groups on the surface [44]. (See Figure 4). Then, the acrylic acid-modified PU stent (PU-AAc) is incubated with EDC/NHS as a coupling agent to create an NHS ester that is substantially more stable than the O-acylisourea intermediate [44]. The dip-coating procedure was conducted using three different concentrations (5, 10, and 20 mg/mL) of CS-FA derivatives; however, the concentration of 20 mg/mL CS-FA derivatives solutions was not able to be used as a coating solution due to its high viscosity and the low solubility of CS-FA derivatives in mild aqueous conditions. The presence and efficacy of coatings were confirmed by crystal violet staining and the XPS technique. Crystal violet dye is an effective adsorbent to CS, and thus a purple color can be seen in the presence of CS [45]. In Figure 5, an increase in purple color intensity is observed with an increased concentration of CS-FA derivatives.

**Figure 6:**
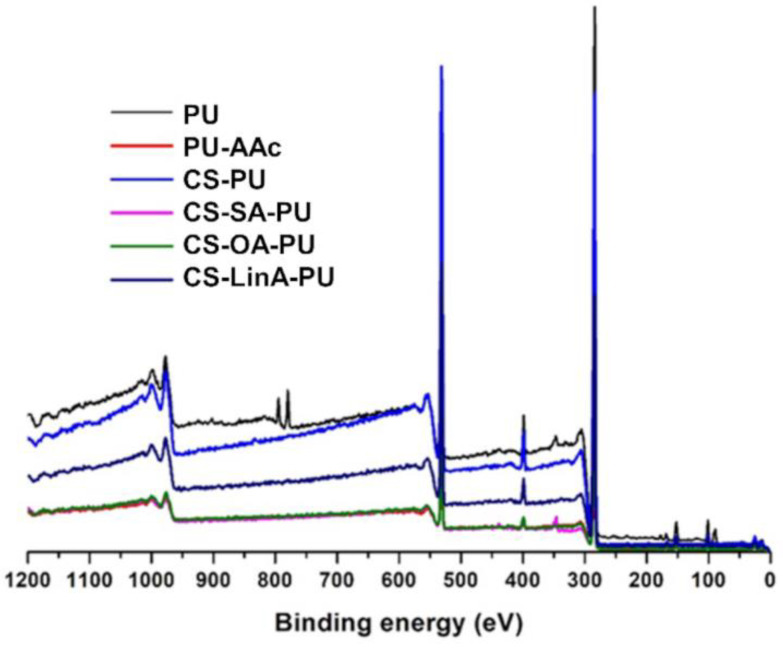
X-ray photoelectron spectroscopy wide scan of untreated and coated PU stents.

**Figure 7:**
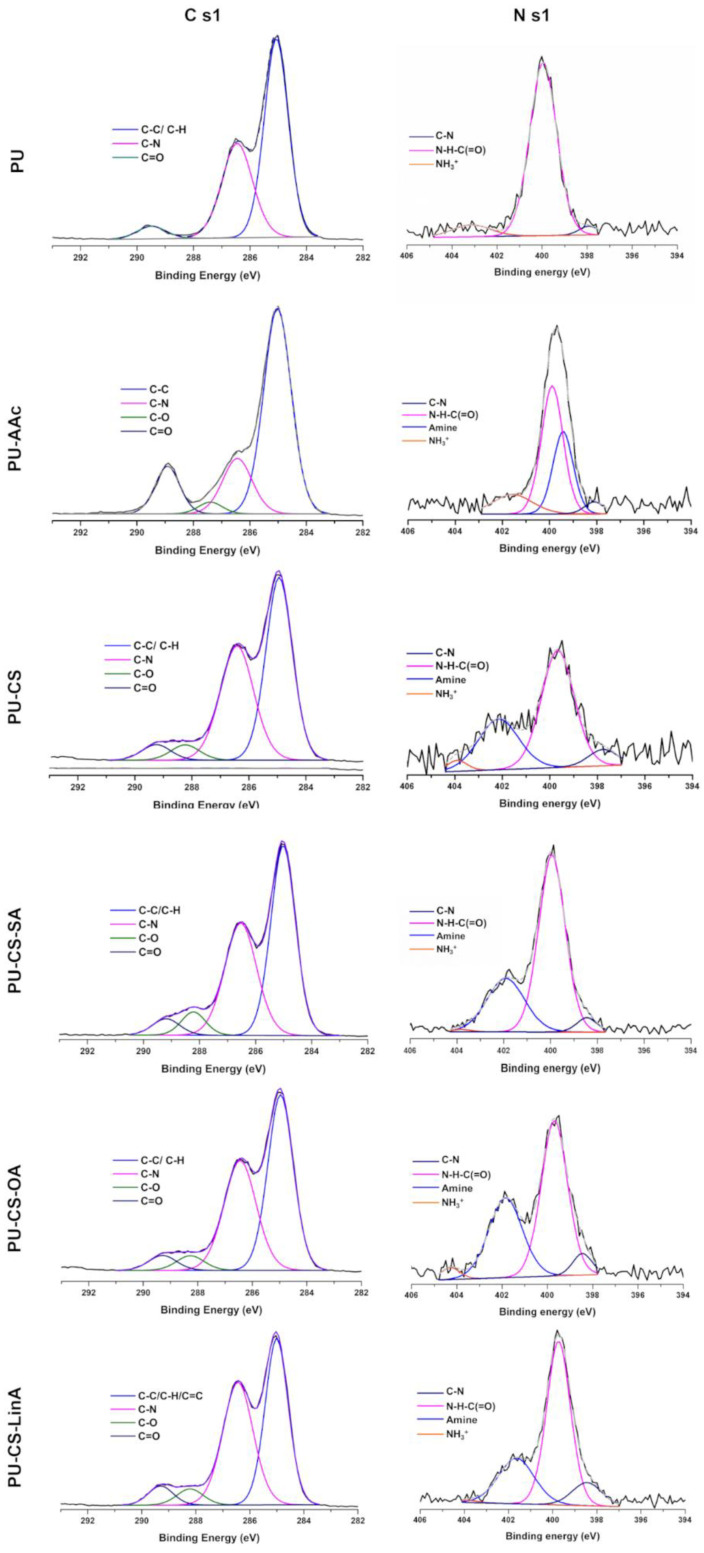
X-ray photoelectron spectroscopy C 1s and N 1s region scans of untreated and coated PU stents.

XPS spectral analysis was employed to verify the activation step and the presence of coatings (Figure 6 and Figure 7). According to the achieved data, the three major composing elements, carbon, oxygen, and nitrogen, were detected on the surface of all PU stents (Figure 6); the summary of the atomic composition of the analyzed surfaces of all coated, PU-AAc, and untreated PU stents, included in the Table S1, are compiled in Table 1.

All the coated PU stents present similar content of N, C and O, as the FA derivatives used for the CS substitution have the comparable C/O ratios; the introduced FA moieties do not significantly influence the chemical composition of the polymeric coating and, consequently, the coated PU stent surface. Moreover, similarly to what has been earlier reported using different coatings with modified CS onto different substrates, the quantity of nitrogen ascertained on the coated surface (∼3–5 at.%) is considerably lower than that observed for CS or the synthesized CS-FA derivatives (∼7–8 at.%) (data not shown) [20]. In this study, despite the similar compositions of CS and CS-FA derivatives-based coatings, a slight variation was noted in the C/O and C/N ratios. The noted reduction in the C/O ratio to values closer to the ones registered for CS (data not shown) confirmed the existence of a CS superficial coating layer over the PU stent. In parallel, the slight increase observed for the C/N ratio suggests that the interactions established between the PU surface and the CS-based coating predominantly occur through a covalent interaction between the amine group of CS and the carboxyl group of the activated PU. The polymeric chain suffers a steric rearrangement by becoming located on the top of the binding covering the amine group.

The high-resolution C 1s spectra with the binding energy ranging from 282 to 293 eV can be fitted into three peaks for PU and four distinct peaks for activated and coated PU stents, as shown in Figure 7. The untreated PU stents C 1s spectra were deconvoluted into three prominent peaks at: 285.1 eV for C-C, C-H, 286.4 eV for C-N, and 289.5 eV for C=O [46,47,48,49]. Following the activation and coating processes, PU-AAc and all CS-FA-PU stents revealed additional bands at 287.4 and 288.2 eV, respectively, which is ascribed to C-O bonding. The achievement of the activation step led to an increase in C=O and C-O elemental contributions due to the enrichment of the PU surface with carboxylic groups [46]. This observation was also confirmed by the appearance of the amine group on the PU-AAc surface in the N 1s spectra (Figure 7 and Table 1). 

Regarding the coating with CS and CS-FA derivatives on the PU surface, the effectiveness of this strategy was evidenced by the increase in the amount of amine bindings and C-N bindings. Herein, it is worth noting that the absence of C-O binding in untreated PU stents can be correlated to the reduced sampling depth during XPS measurements (nearly 5 nm depth) and the variability of molecular conformation or organization, which is in agreement with previously reported studies [20,50]. By examining the contribution of C-N binding in C 1s spectra, the one from PU-AAc showed a decrease in the relative quantity of C-N bonding (from 27.12% to 21.54%), which is due to the increase in the amount of carboxyl groups at the activated PU-stent surface. Following the coating process, the rise in the number of C-N bindings confirms the presence of CS (GlcNac and GlcN units). Remarkably, compared to that of untreated PU, the CS-SA-PU exhibited a reduced quantity in C-N bonding, while for CS-OA-PU and CS-LinA-PU, this quantity was raised. This observation may be related to the conformational changes that can be induced among the molecules that form the coating, mainly due to the presence of fewer sterically hindered and highly mobile SA moieties (long aliphatic chain) oriented towards the outer part of the coating masking the amine group that is under it [51]. In addition, the increasing number of double bonds in the unsaturated FA moieties caused an increase in steric hindrances, which restricts the molecular mobility and allows for the detection of the C-N bindings on the coated sample’s surface [52,53].

The deconvoluted N 1s spectra of PU offer three typical peaks around 398.5, 400.2, and 402.6 eV, attributed to C-N, NH-C(=O)-, and -NH_3_^+^ bonds [54,55], respectively, as shown in Figure 7. In all N s1 spectra of the samples, the low-intensity peaks at 398.5 eV can be correlated to sp3 C–N bonding [54]. In addition, unlike the absence of the amine group in the N 1s spectra of PU, it is possible to observe for PU-AAc and all the coated PU stents additional peaks at 399.5 and 401.8 eV, respectively, attributed to amine groups. The peak observed for the PU-AAc sample (at 399.5 eV) can be assigned to the amino groups resulting from the degradation of amide linkages in the activation step [56,57]. By the coating process, a shift of this peak to 401.8 eV is observed that may be due to the existence of primary amine groups from units of ungrafted CS with FA derivatives in the coated PU stents [58,59]. 

In analyzing the contribution of urethane groups to the coating process, it was observed that the number of urethane bindings diminished and slightly shifted the binding energy from 400.2 eV to 399.7 eV. This result suggests a reduction in the quantity of urethane groups due to the interaction between CS and PU, as expected, and also, due to the masking effect of the aliphatic chains located over it. On the other hand, compared to the other nitrogen bindings (C-N, urethane, and amine), the -NH_3_^+^ peak is observed at higher binding energy values (402.6 or 403.8 eV) because of the charge effect. Different quantities of the -NH_3_^+^ bindings were determined in each sample since the organization and/or conformation of the molecules in the coatings is influenced by the high vacuum during XPS analyses [51]. Overall, the appearance of an amine band in the N 1s region is clear, due to PU stent surface characteristics of the CS and its derivatives and consequent reduction of the urethane band related to the PU stent, confirming the existence of a superficial coating. The ratio between the amine/urethane bands is, as expected, similar for all the coated PU tubes, due to the analogous chemical composition of the employed polymeric coatings. Similar behavior has been earlier reported for PEI chains covalently attached to PU surfaces, where besides the urethane peak observed at around 400 eV attributed to the backbone of aliphatic polymer structures, an additional peak in N1s spectra is present, indicating the amine moiety from the PEI attached to the PU substrate [51].

**Figure 8:**
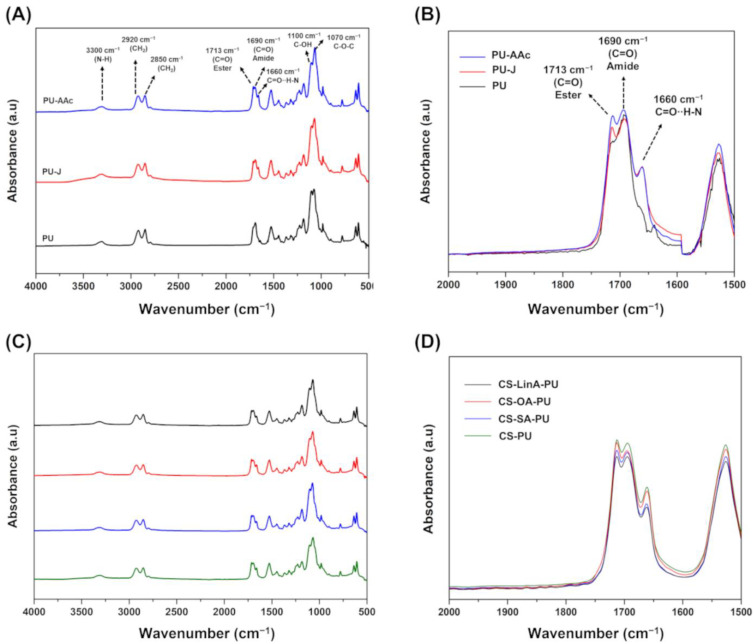
FTIR-ATR spectra of untreated PU stent, PU stent treated by Jone’s reagent (PU-J), acrylic acid-modified PU stent (PU-AAc) (**A**); magnified region from 2000 cm^−1^ to 1500 cm^−1^ of untreated PU stent, PU-J and PU-AAc (**B**); CS and CS-FA coated-PU stents (**C**); magnified region from 2000 cm^−1^ to 1500 cm^−1^ of CS and CS-FA coated-PU stents (**D**).

FTIR-ATR measurements were performed on the produced PU stent, as well as on the coated PU stents, in order to identify the degree of achievement of the activation and coating strategies in the entire specimen. The expected structure of untreated PU stents is shown by the typical absorption bands at 3300, 2920, and 2850 cm^−1^ attributed to N-H stretching vibration, and the asymmetric and symmetric C–H stretching vibrations, respectively [60]. The absorbance band at 1690 cm^−1^ is assigned to C=O stretching vibration in the amide group, while the peak present at 1713 cm^−1^ is attributed to the stretching vibration of C=O in the ester group and/or carboxyl group [61]. Upon the activation step, the increasing absorption band at 1713 cm^−1^ contributes to confirming the existence of the carboxyl group. Moreover, as illustrated in detail by Figure 8B, a new absorbance band at 1660 cm^−1^ is attributed to the interaction between hydrogen and carbonyl group [62,63]. Additionally, the increasing absorbance bands at 1100 and 1070 cm^−1^, assigned to C-OH and C-O groups, further verified that the PU stent was enriched by the carboxyl groups [64]. On the other hand, the FTIR-ATR spectra of CS-FA derivatives-coated PU stents exhibited similar bands to the spectra of activated PU stents (Figure 8C,D). As aforementioned, the coating process is performed by the interaction between the amino group in CS-FA derivatives with the active ester group in PU stents. Therefore it is not possible to interpret it as evidence that the PU surface is coated by CS and CS-FA derivatives since the amide stretching bands (O=C-N-H) in the coated PU stents were overlapped by the amide absorption bands in the PU structure [65].

**Figure 9:**
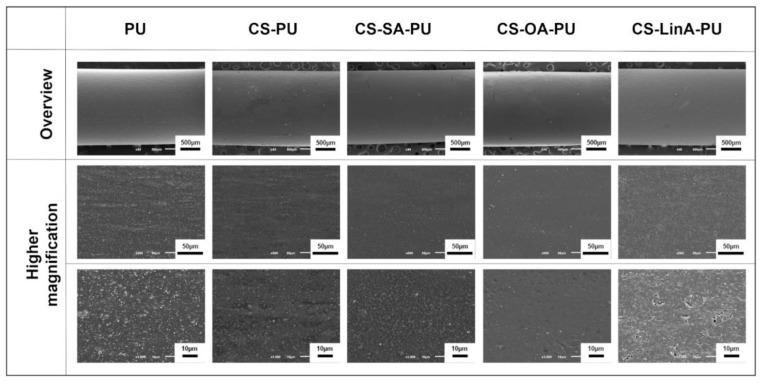
Representative SEM micrographs of the outer surface of untreated PU stent (control), CS-PU, CS-SA-PU, CS-OA-PU and CS-LinA-PU. The scale bars are 500 µm, 50 µm, and 10 µm from top to bottom.

All the formulations were further examined by SEM microscopy to evaluate the morphology of the coated PU stents. Figure 9 presents the top-view SEM micrographs of the outer surface of the untreated PU stent and the coated PU stents with CS-FA derivatives. The untreated PU stent had a relatively rough surface morphology. CS and CS-FA coated PU stents also presented a homogeneous morphology, revealing a uniform distribution of the coating. Based on the top-view SEM micrographs of the coated PU stents, no cracks or any apparent defect were observed. However, the higher magnifications of SEM micrographs revealed distinct surface morphology for each CS-FA-PU derivative. Similar morphological alterations were reported in a previous study for hydroxypropyl-methylcellulose (HPMC) based films containing diverse saturated and unsaturated FA derivatives. The differences in distinct shapes and sizes were observed on the surface of these films due to the presence of FA derivatives, in contrast to the homogeneous structure of the control film [66].

As is shown easily in Figure 9, the surface of the OA-CS-PU stent was remarkably smooth, the surface of CS-SA-PU and CS-PU stents was on the contrary rougher. These alterations in the roughness of the coatings can be attributed to the differences in the hydrophilicity of the CS and CS-FA derivatives. Clearly, the untreated PU stent is known to be essentially hydrophobic. However, after the activation treatment, its chemical structure alters, and the surface roughness was affected by the hydrophilicity of coating materials. Accordingly, previous studies suggested that surface roughness increases due to increased hydrophilicity. On the other hand, as reported previously, individual FA derivatives exhibit higher hydrophobicity when the alkyl chain is longer in the molecule and less hydrophobicity as the unsaturation degree increases. Therefore, it can be likely interpreted that CS-OA coatings have a smoother surface due to their high hydrophobicity [67].

Furthermore, the surface of CS-LinA-PU exhibited a porous and glossier structure. This structure can be interpreted as the existence of branched CS molecules and non-linear LinA, with the formation of notable irregular protrusions resulting in their mutual entanglement, which is consistent with results obtained in previous studies [66]. Consequently, the surface of the untreated PU stent was converted to a relatively less rough and porous surface by CS-FA coatings, which is a noteworthy feature for preventing bacterial attachment since bacteria tends to accumulate in surface cavities to augment the contact site with the material in order to protect itself from environmental damages [68]. However, it should be noted that bacterial attachment can be affected by various parameters such as surface roughness, hydrophobicity, environmental factors, and/or surface charge, etc. [69].

### 3.3. Biological Performance

#### 3.3.1. Cytotoxicity Studies against L929 Murine Fibroblasts

**Figure 10:**
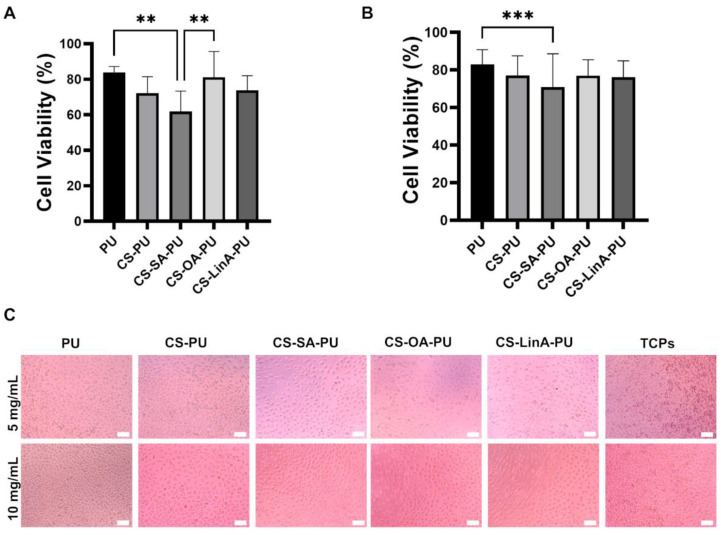
L929 cell viability after exposure to 24 h extracts of PU stents and coated PU stents (**A**) 5 mg/mL. (**B**) 10 mg/mL. Results were expressed as the mean ± standard deviation values of three independent assays conducted in triplicate. ***, *p*-value ≤ 0.001; **, *p*-value ≤ 0.01. (**C**) Cell morphology after exposure to 24 h extracts. The scale bar is 100 µm.

Observing the obtained cellular viability, it is possible to verify that 24 h extracts from both coating concentrations display similar toxicity. Furthermore, for both the highest and lowest used concentration of coating solutions (5 and 10 mg/mL, respectively), the cytotoxicity of coated PU stents, except for CS-SA-PU, does not differ significantly from that of uncoated PU stents, suggesting the biocompatibility of the applied coatings. However, in both concentrations, CS-SA-PU led to a slight decrease in cell viability compared to the control PU stent. It has not been possible to provide definite answers for why the concentration of 5 mg/mL CS-SA coating solution induced lower viability than the higher concentration of 10 mg/mL. This difference can presumably be correlated with the complicated relationship between the concentration of CS-FA derivatives and their behavior in coatings and, accordingly, their interaction with biological systems [70]. On the other hand, cytotoxicity results are further supported by the cellular morphology observed for each individual coating, where a well-stretched morphology with good anchorage to the well surface can be observed (Figure 10C). Since no significant effect was seen on cellular viability and to guarantee a more prominent antibacterial effect, coatings of 10 mg/mL were selected to carry out the assays pertaining to antibacterial activity and bacterial adhesion prevention.

#### 3.3.2. Antibacterial Assessments

**Figure 11:**
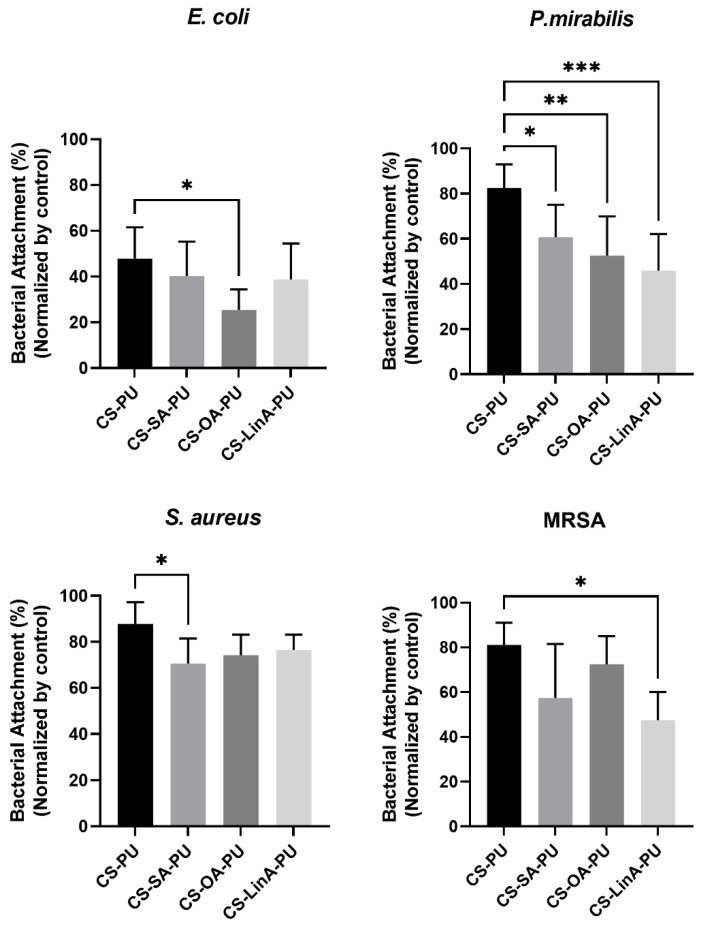
Percentage of adhered bacteria on the surface of CS-PU, CS-SA-PU, CS-OA-PU, and CS-LinA-PU upon 24 h exposure for each selected bacterial strain. The bacterial attachment percentage was ascertained utilizing the AB kit and normalized with control PU stent. Results were expressed as the mean ± standard deviation values of six independent assays conducted in triplicate. ***, *p*-value ≤ 0.001; **, *p*-value ≤ 0.01. *, *p*-value ≤ 0.05; ns, *p*-value > 0.05 (ns, no significant).

In order to investigate the antimicrobial potency of the obtained coatings, we evaluated bacterial attachment on the surface of coated PU stents by Gram-negative bacteria (*Proteus Mirabilis*-*P. mirabilis* and *Escherichia coli*-*E. coli*) and Gram-positive bacteria (*Staphylococcus aureus*-*S. aureus* and *methicillin-resistant Staphylococcus aureus*-MRSA), which are responsible for common stent-related complications. The results deduced via the Alamar blue (AB) assay following biofilm exposure to coated PU stents with CS and CS-FA derivatives normalized by untreated PU stents against the selected bacterial strains following respective times are presented in Figure 11. Analyzing these data, it can be easily seen that CS-FA derivatives-coated PU stents exhibited stronger activity against Gram-negative bacteria than Gram-positive bacteria. Following other reported studies, Gram-negative bacteria are generally more susceptible to the antibacterial effects of CS due to its more hydrophilic membrane structure and, hence, higher surface polarity [71,72]. Many studies have reported the presence of lipopolysaccharides containing phosphate and pyrophosphate groups on the outer membrane of Gram-negative bacteria such as *E. coli* and *P. mirabilis*, which increases the negative charge density on the surface [73]. These findings heavily point out the establishment of interactions between positively charged CS molecules and negatively charged bacterial cell membranes, corroborating the obtained results. On the other hand, the more effective antimicrobial activity of CS-FA-coated PU stents can be associated principally with the presence of hydrophobic tails of FA derivatives, which may disrupt the outer membrane of Gram-negative bacteria cells, causing leakage of cellular contents and eventually leading to bacterial death [74].

**Figure 12:**
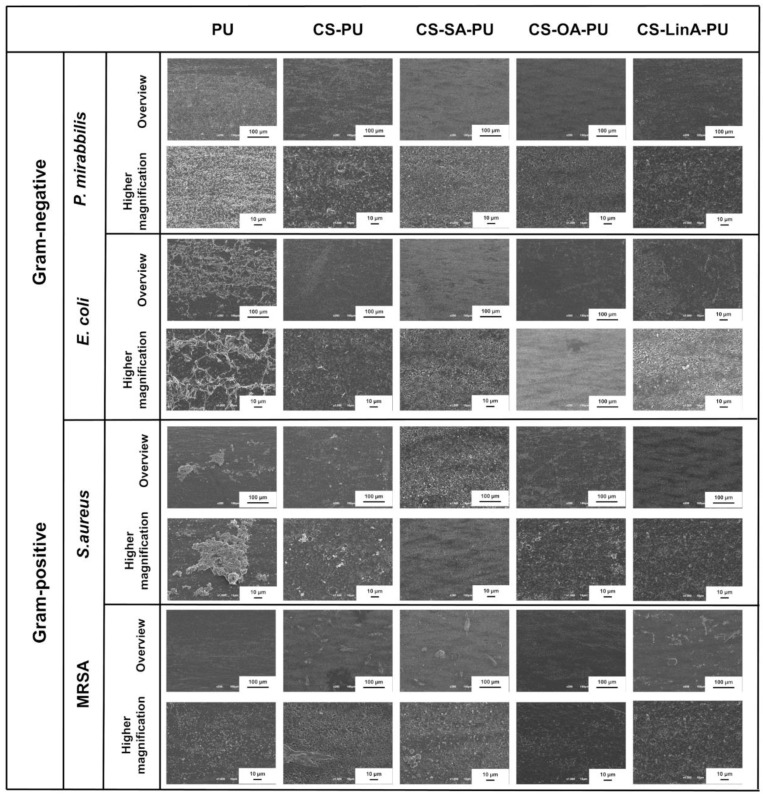
Representative SEM micrographs of *P. mirabilis*, *E. coli*, *S. aureus* and *MRSA* on the outer surface of control PU stents, CS-PU, CS-SA-PU, CS-OA-PU and CS-LinA-PU.

To further validate the developed coatings and the obtained results in the metabolic activity-based AB assay, SEM analysis was utilized to examine the bacterial colonization at 24 h for each bacterial strain on the outer surface of untreated PU stents and the coated PU stents with CS and CS-FA derivatives with the results are shown in Figure 12. Notably, bacterial attachment of all selected strains on the surface of coated PU stents with CS-FA derivatives decreased compared to untreated PU stents and CS-coated PU stents. Considering the AB results, the SEM micrographs corroborate the fact that CS-FA-based coatings are more effective on Gram-negative bacteria for the aforementioned reasons. Furthermore, the gathered data indicate that the base material (untreated PU stent- proprietary material) already performs a distinguished efficiency against Gram-positive bacteria, including *S. aureus* and MRSA. Hence, CS-FA derivates-based-coatings slightly prevented bacterial attachment against the tested Gram-positive bacteria. On the other hand, the performance of the coated PU stents against Gram-negative bacteria was notably evolved with the developed materials and surface functionalization approach.

**Figure 13:**
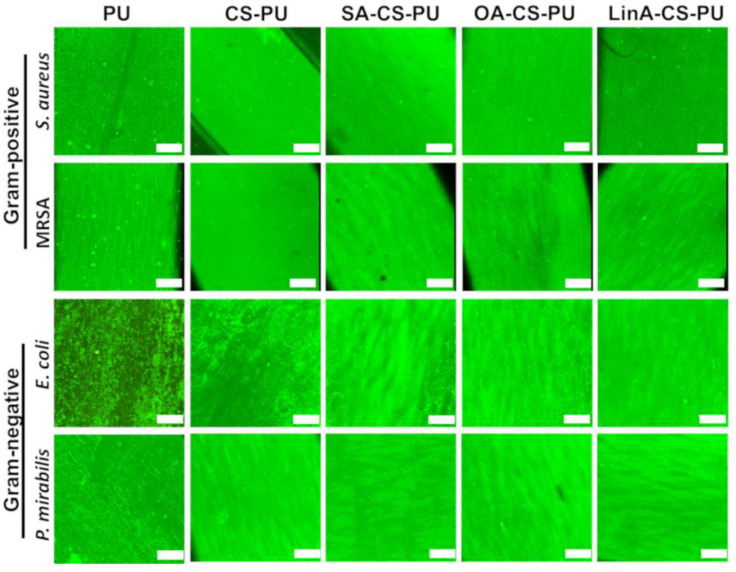
Live/dead fluorescence assay of *E. coli*, *P. mirabilis*, MRSA and *S. aureus*: Images of the outer surface of control PU stent, CS-PU, CS-SA-PU, CS-OA-PU and CS-LinA-PU. Scale bar is 100 µm.

Additionally, the viable bacteria adhering to the surface of CS and CS-FA derivatives-coated PU stents were further confirmed by analysis of confocal images following a Live/dead fluorescence assay against the selected microbial strains. Thus, the obtained data lend support to qualitative and quantitative conclusions that the CS-FA derivatives-coated PU stents exhibit antibacterial activity associated with the SEM micrographs and AB assay. As shown in Figure 13, for each tested bacterial strain, the number of viable adherent bacteria on the surface of all coated PU stents was significantly lower than that on the untreated PU stent. As a control for autofluorescence of the samples, non-coated and coated stents were also incubated with LIVE/DEAD^®^ BacLight^TM^—data are presented in Figure S2.

Considering the limitations of the presently available ureteral stents and the versatile promising properties of CS and FA derivatives, an effective antibacterial coating based on CS-FA derivatives was developed using a straightforward and effective surface functionalization method. The obtained results show that these coated materials can prevent bacterial attachment and hence colonization of all tested microorganisms, even for Gram-positive bacteria, via distinct mechanisms. This study provides strong prospective evidence for the potential of coatings based on CS grafted with saturated and unsaturated FAs to be wide-spectrum antimicrobial agents that, if suitably offered, can be utilized in indwelling medical devices as an effective therapeutic alternative against microbial infections.

## 4. Conclusions

Combating bacterial infections associated with ureteral stents has become an enormous challenge due to exposure to complex microbial communities. Moreover, along with the emergence of multidrug-resistant bacterial strains, new bio-compatible therapeutic agents that do not incur bacterial resistance are needed to diminish the spread of bacterial infections. For this purpose, natural compounds are exciting and promising candidates as coating agents for use by current strategies. Prior work has documented the biocompatibility and amphiphilic properties of CS-FA derivatives-based biopolymer for several applications such as wound dressing, nonviral gene delivery vectors, and multifunctional coating materials. For instance, the antibacterial activity of the developed coatings containing CS grafted with three different unsaturated FA derivatives was tested against *E. coli* bacteria. Other recent studies have focused on structural characterization of CS-FA derivatives or investigated the antibacterial activity against a particular bacterial strain. 

In this work, we successfully obtained formulations of CS modified with either one saturated or two distinct unsaturated FA derivatives via a carbodiimide-mediated coupling reaction. Subsequently, PU stents were coated with synthesized CS-FA derivatives through covalent interactions, following an effective activation step. The optimum concentration of the coating solutions was determined by cytotoxicity studies against L929 murine fibroblasts, confirming that a concentration of 10 mg/mL exhibited negligible cytotoxicity. In addition, the efficiency of preventing the bacterial attachment of Gram-positive and Gram-negative bacteria, including *S. aureus*, MRSA, *E. coli*, and *P. mirabilis* strains, was evaluated by AB assay collaborating with SEM micrographs. Obtained data revealed the more effective performance of all CS-FA derivatives-coated-PU stents against Gram-negative compared to Gram-positive bacteria. The viable bacteria adhering to the surface of all coated PU stents were further assessed by analysis of confocal images following Live/dead fluorescence assay against the selected microbial strains. The results confirmed that the number of viable adherent bacteria on the surface of all coated PU stents was significantly lower than on the untreated PU stent, which is consistent with the results gathered from SEM micrographs and the AB assay.

In summary, these CS-FA conjugates represent a novel class of highly effective biopolymers and hence, may serve as antibacterial agents in various fields of biomedicine, agriculture, food processing, etc. Furthermore, the CS-FA derivatives were functionalized in PU stents by the effective and straightforward coating method used. All PU stents coated with CS-FA derivatives showed bacterial anti-adhesive effects and biocompatibility, suggesting that it could exhibit other synergistic effects such as the anti-encrustation activity triggered by bacterial adhesion on the surface of medical devices. Therefore, future studies should focus on investigating the stability and antibacterial and anti-encrustation performance of CS-FA derivatives-based coatings under dynamic conditions to mimic the urine environment. In addition, further screening using other organisms involved in urinary tract infections and human urethral cell lines is suggested. Consequently, the current findings indicate that CS-FA derivatives may serve as an antimicrobial coating agent, which can be applied to other medical devices as well as ureteral stents.

## Figures and Tables

**Table 1:** Percentage contributions of distinctive chemical bonds in the high-resolution C 1s and N 1s spectra.

**C 1s Assignment (%)**						
**Components**	PU	PU-AAc	CS-PU	CS-SA-PU	CS-OA-PU	CS-LinA-PU
**C-C/C-H/C=C**	49.05 ± 1.31 (285.1 eV)	45.63 ± 3.81 (284.9 eV)	37.73 ± 1.64 (285.0 eV)	39.11 ± 3.16 (284.9 eV)	31.31 ± 5.13 (284.9 eV)	34.69 ± 1.60 (284.9 eV)
**C-N**	27.13 ± 1.20 (286.4 eV)	21.54± 7.22 (286.4 eV)	29.51 ± 6.94 (286.4 eV)	25.85 ± 1.26 (286.4 eV)	27.33 ± 4.02 (286.4 eV)	30.02 ± 1.12 (286.4 eV)
**C=O**	3.53 ± 1.13 (289.5 eV)	7.47± 2.77 (289.0 eV)	6.49 ± 4.30 (289.0 eV)	3.05 ± 0.58 (289.2 eV)	4.82 ± 0.55 (289.2 eV)	3.37 ± 0.62 (289.2 eV)
**C-O**	-	2.47± 0.08 (287.4 eV)	3.22 ± 0.10 (288.2 eV)	3.43 ± 0.88 (288.2 eV)	5.66 ± 1.85 (288.2 eV)	3.65 ± 0.42 (288.2 eV)
**N 1s Assignment** (**%**)						
**C–N**	0.13 ± 0.02 (398.5 eV)	0.13 ± 0.01 (398.5 eV)	0.17 ± 0.04 (398.5 eV)	0.15 ± 0.01 (398.5 eV)	0.2 7± 0.01 (398.5 eV)	0.45 ± 0.04 (398.5 eV)
**Urethane** **-N-C**(**=O**)	2.86 ± 0.05 (400.2 eV)	1.05 ± 0.55 (399.7 eV)	0.86 ± 0.36 (399.7 eV)	1.16 ± 0.12 (399.7 eV)	1.94 ± 0.31 (399.7 eV)	0.97 ± 0.09 (399.7 eV)
**NH_3_^+^**	0.32 ± 0.02 (402.6 eV)	0.25 ± 0.02 (403.8 eV)	0.05 ± 0.01 (403.8 eV)	0.11 ± 0.09 (403.8 eV)	0.15 ± 0.01 (402.6 eV)	0.03 ± 0.01 (403.8 eV)
**Amine**	-	0.94 ± 0.01 (399.5 eV)	1.66 ± 0.72 (401.8 eV)	2.30 ± 0.44 (401.8 eV)	2.05 ± 0.77 (401.8 eV)	2.74 ± 0.16 (401.8 eV)

## Data Availability

Not applicable.

## Supplementary Materials

The following supporting information can be downloaded at: https://www.mdpi.com/article/10.3390/ma15051676/s1, Figure S1: DSC thermograms for powder of commercial CS and synthesized CS-FA derivatives); Figure S2: Live/dead fluorescence assay performed on all formulations to infer background effect of the counterparts; Table S1: Elemental composition (% C, N, O) of untreated PU tube, acrylic acid-modified PU tube (PU-AAc) and CS-FA derivatives coated PU tubes.
