# Supplementary material for: Surface Functionalization of Ureteral Stents-Based Polyurethane: Engineering Antibacterial Coatings
Source: Materials (Basel). 2022 Feb 23;15(5):1676. doi: 10.3390/ma15051676 (PMC8910958; doi:10.3390/ma15051676)
Supplement: Supplementary file 1 [file materials-15-01676-s001.zip › materials-1555614-supplementary.pdf]

*materials*

MDPI

*Supplementary Materials*

# Surface Functionalization of Ureteral Stents-Based Polyurethane: Engineering Antibacterial Coatings

**Kardelen Ecevit [1,2], Eduardo Silva [1,2], Luísa C. Rodrigues [1,2], Ivo Aroso [1,2], Alexandre A. Barros [1,2], Joana M. Silva [1,2,*] and Rui L. Reis [1,2]**

1 3B's Research Group, I3Bs–Research Institute on Biomaterials, Biodegradables and Biomimetics, University of Minho, Headquarters of the European Institute of Excellence on Tissue Engineering and Regenerative Medicine, Avepark, Parque de Ciência e Tecnologia, Zona Industrial da Gandra, 4805-017 Barco GMR, Portugal; kardelen.ecevit@i3bs.uminho.pt (K.E.); eduardo.silva@I3Bs.uminho.pt (E.S.); luisa.rodrigues@i3bs.uminho.pt (L.C.R.); ivo.aroso@i3bs.uminho.pt (I.A.); ip@i3bs.uminho.pt (A.A.B.); rgreis@i3bs.uminho.pt (R.L.R.)

2 ICVS/3B's PT Government Associated Laboratory, 4805-017 Guimaraes, Portugal

* Correspondence: joana.marques@i3bs.uminho.pt

**Citation:** Ecevit, K.; Silva, E.; Rodrigues, L. C.; Aroso, I.; Barros, A.A.; Silva, J.M.; Reis, R.L. Surface Functionalization of Ureteral Stents-Based Polyurethane: Engineering Antibacterial Coatings. *Materials* **2022**, *15*, 1676. https://doi.org/10.3390/ma15051676

Academic Editor: Anton Nikiforov

Received: 29 December 2021

Accepted: 14 February 2022

Published: 23 February 2022

**Publisher's Note:** MDPI stays neutral with regard to jurisdictional claims in published maps and institutional affiliations.

**Copyright:** © 2022 by the authors. Licensee MDPI, Basel, Switzerland. This article is an open access article distributed under the terms and conditions of the Creative Commons Attribution (CC BY) license (https://creativecommons.org/licenses/by/4.0/).

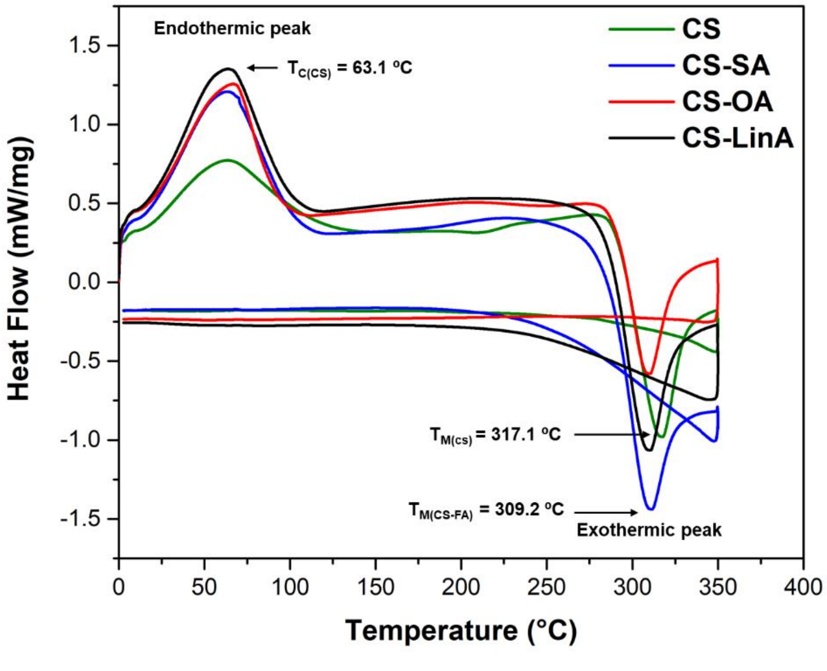


**Figure S1.** DSC thermograms for CS and synthesized CS-FA derivatives.

**Table S1.** Elemental composition (% C, N, O) of untreated PU stent, acrylic acid-modified PU stent (PU-AAc) and CS-FA derivatives coated PU stents.

| | ATOMIC CONCENTRATION [%] | | |
|---|---|---|---|
| | **C** | **O** | **N** |
| **PU** | 79.64 ± 0.24 | 17.02 ± 0.07 | 3.34± 0.01 |
| **PU-AAC** | 75.97 ± 0.66 | 19.80 ± 0.80 | 1.61± 1.5 |
| **CS-PU** | 70.97 ± 2.59 | 24.23 ± 3.32 | 2.93 ± 0.96 |
| **CS-SA-PU** | 71.78 ± 2.16 | 21.74 ± 1.53 | 3.38 ± 0.86 |
| **CS-OA-PU** | 68.29 ± 3.39 | 23.93 ± 2.03 | 4.79 ± 0.15 |
| **CS-LINA-PU** | 71.74 ± 1.01 | 22.15 ± 0.78 | 3.85 ± 0.24 |

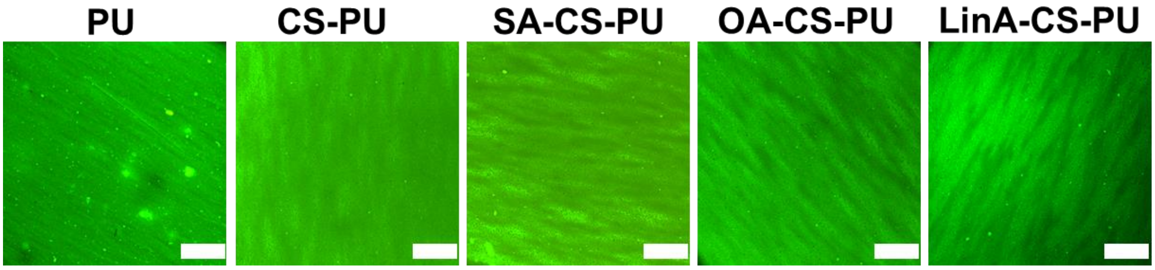


**Figure S2.** Live/dead fluorescence assay performed on all formulations to infer background effect of the counterparts: Images of the outer surface of untreated PU stent (control), CS-PU, CS-SA-PU, CS-OA-PU and CS-LinA-PU. Scale bar is 100 μm.
